# Huge Bilateral Ovarian Cysts With Concurrent Hypothyroidism: A Case Report

**DOI:** 10.7759/cureus.58837

**Published:** 2024-04-23

**Authors:** Deepti Jain, Saksham Jain

**Affiliations:** 1 Obstetrics and Gynecology, Chotu Ram Hospital, Rohtak, IND; 2 Radiodiagnosis, Dr D.Y. Patil Medical College, Hospital and Research Centre, Pune, IND

**Keywords:** ovarian hyperstimulation, enlarged ovaries, multicystic, hypothyroidism, ovarian cyst

## Abstract

A 19-year-old girl with a short stature and presenting low intelligence quotient, illegible speech, and a greatly distended abdomen was seen at the gynecological outpatient department. She underwent investigation and was found to have large abdominopelvic multicystic ovaries with no malignant features and CA125 levels within the normal range for premenopausal women. Her thyroid-stimulating hormone (TSH) was markedly elevated. She received a diagnosis of untreated severe hypothyroidism with benign giant ovarian cysts, posing a grave risk of cyst rupture and imminent complications. The parents were counseled, and they accepted the risk, agreeing to conservative therapy. Levothyroxine replacement therapy was initiated, and after one month, her TSH levels normalized. Follow-up ultrasonography after one month of her therapy revealed a marked decrease in ovarian cyst size. Thyroid replacement therapy was continued, and at the end of three months, the cysts disappeared, and the ovaries, much smaller, showed polycystic ovarian morphology. Careful analysis of clinical signs, investigations, and appropriate therapy helped avoid unnecessary surgery.

## Introduction

Hypothyroidism, if untreated or inadequately treated, can have a devastating impact on the physical growth, endocrino-metabolic function, and reproductive health of a woman. Additionally, it results in the impairment of neurocognition and can cause severe mental retardation in affected patients. Ovarian cysts associated with hypothyroidism are not very common. However, individual case reports and case studies have been documented to raise awareness regarding the nature of ovarian cysts and the therapies used to treat them. Treesa et al. have published a case series involving three patients with enlarged ovaries and hypothyroidism, which mimicked malignancy [1].

## Case presentation

A 19-year-old girl presented to the outpatient clinic with her parents. They expressed concern about her increasing abdominal size and frequent episodes of pain. Additionally, they noted her speech was illegible and her behavior seemed more childish and immature than expected for her age. The parents informed the medical staff that she had experienced occasional menstruation over the past few years, with a scanty flow. Informed consent was obtained from the girl's parents for conducting the examination and performing relevant investigations.

On examination, she was 150 cm tall, weighed 66 kg, and had a body mass index of 29.3 kg/m². She had a puffy, rounded face, mild pallor, distended abdomen, and showed no signs of clinical hyperandrogenism. There was no visible pigmentation anywhere on her body. During the abdominal examination, a diffuse, large, soft mass extending approximately 10 cm above the symphysis pubis and measuring 14 cm transversely, with no clearly defined margins, was palpable. Per vaginum examination was omitted due to the patient's unmarried status. Investigations and endocrine assays were performed to delineate the diagnosis.

**Figure 1:**
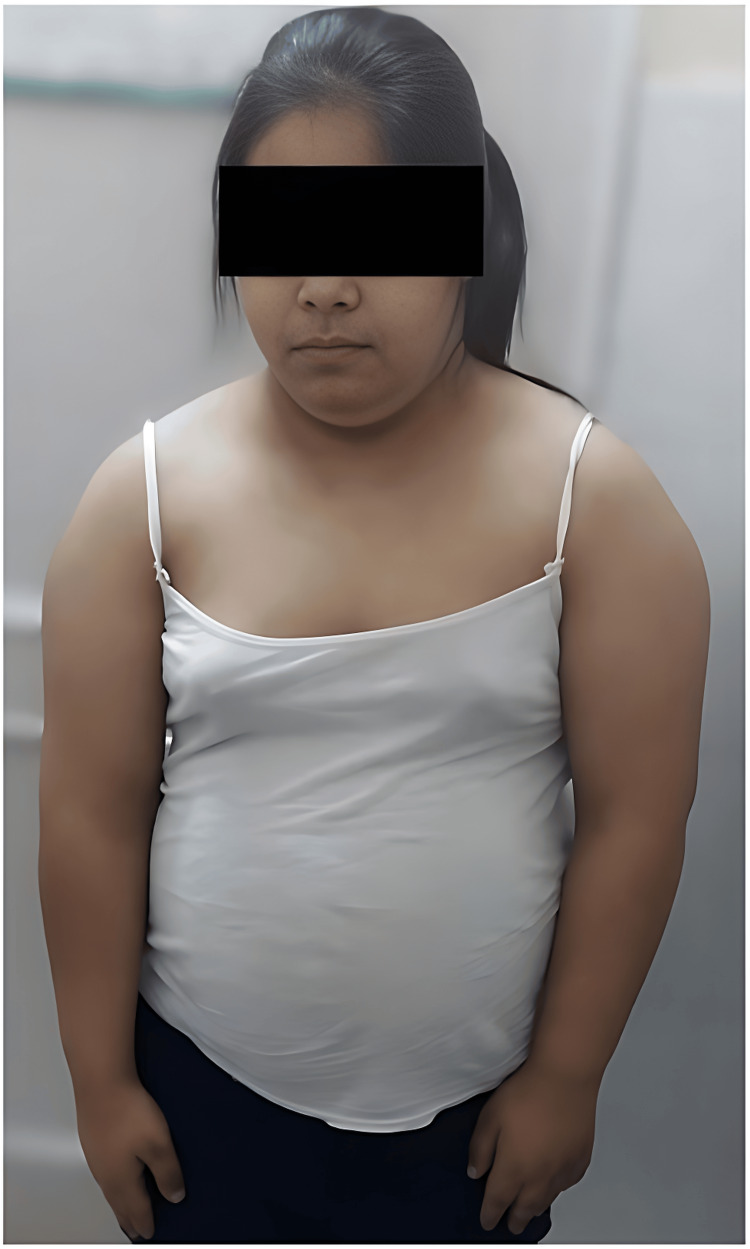
A 19-year-old girl with a rounded, puffy face, exhibiting mild pallor and a protuberant abdomen.

Figure 1 depicts an image of the 19-year-old girl with a rounded, puffy face, exhibiting mild pallor, and a protuberant abdomen.

Laboratory and endocrine investigations, as tabulated in Table 1, revealed reduced hemoglobin, impaired glucose tolerance, elevated serum prolactin level, elevated thyroid-stimulating hormone level (TSH), reduced luteinizing hormone (LH) levels, and reduced follicle-stimulating hormone (FSH) levels.

**Table 1:** Laboratory and endocrine assays of the patient.

Parameter	Patient Value	Normal Range
Hemoglobin	11.5 gm%	>12gm%
Blood Sugar Fasting	110mg%	70-100mg%
CA-125	46.5IU	<50IU (Premenopausal female)
Serum Glutamic Oxaloacetic Transaminase (SGOT)	14.14IU/L	0-40IU/L
Serum Glutamate Pyruvate Transaminase (SGPT)	10.61IU/l	0-40 IU/L
Serum Creatinine	1.13mg%	0.4-1.4mg%
Total leucocyte count	6080/cu mm	4000-11000/cu mm
Thyroid stimulating hormone	>200mIU/ml	0.52-4.30mIU/ml
Follicle stimulating hormone	0.30mIU/ml	3.5-12.5mIU/ml (Follicular phase)
Luteinizing Hormone	<0.10mIU/ml	2.4-12.6mIU/ml (Follicular phase)
Serum Prolactin	46.68ng/ml	4.79-23.3 ng/ml (Follicular phase)

**Figure 2:**
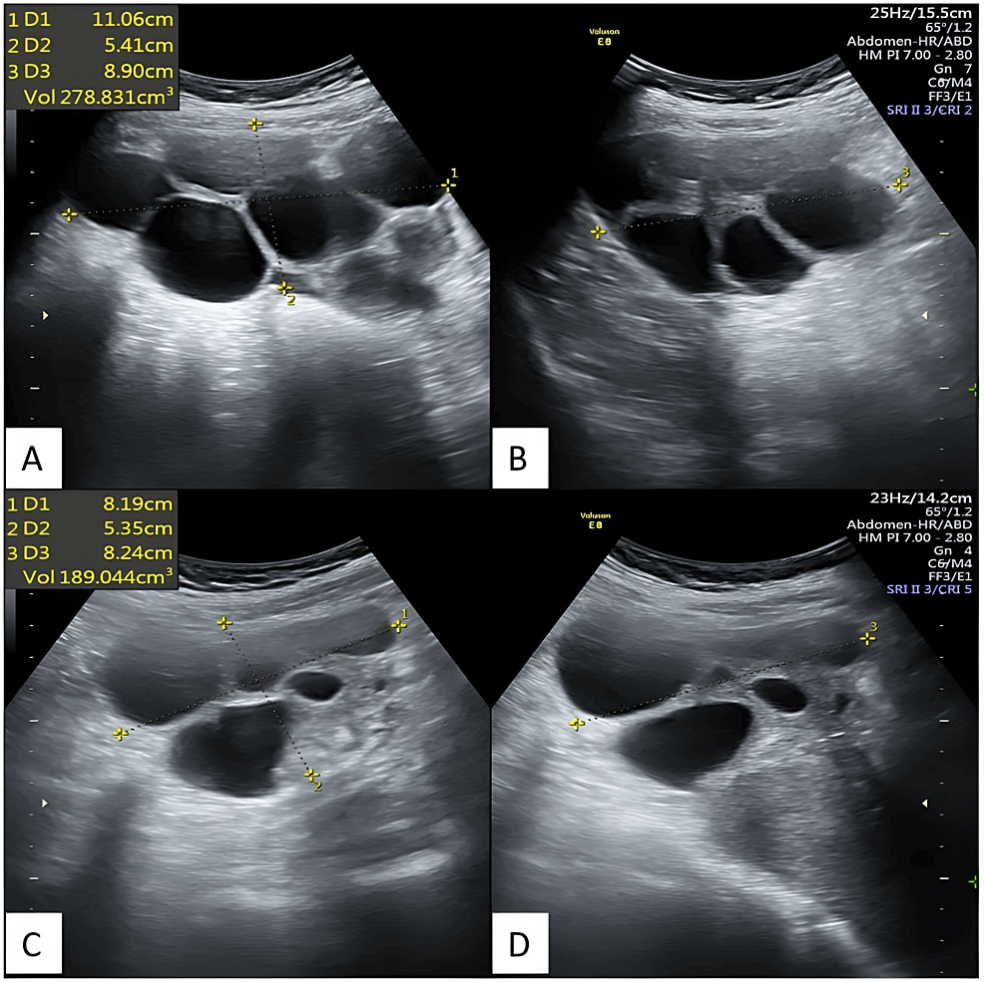
Transabdominal ultrasonography images depict enlarged bilateral ovaries with multiple cysts Transabdominal ultrasonography depicts an enlarged right ovary in axial (A) and sagittal (B) sections, and an enlarged left ovary in axial (C) and sagittal (D) sections, both exhibiting multiple cysts.

Transabdominal sonography (TAS) was performed to determine the nature of the mass felt on per-abdominal examination. The TAS revealed a uterus of normal size and echotexture, with an endometrial thickness of 8 mm. The right ovary measured 110x54x89 mm (278 cc), while the left ovary measured 81x53x82 mm (189 cc). Both ovaries were bulky and contained multiple round-oval anechoic lesions of varying sizes, without any evidence of septa, internal echoes, or internal vascularity, suggestive of simple ovarian cysts. The largest cyst measured 41x42 mm in the right ovary and 37x35 mm in the left ovary. No ascites, enlarged lymph nodes, or pleural effusion were observed. Figure 2 depicts transabdominal ultrasonography images showing enlarged bilateral ovaries with multiple cysts.

A radiological diagnosis of benign functional ovarian cysts was suggested. Subsequent investigations revealed normal CA125 levels, mild anemia, impaired glucose tolerance, normal hepatic and renal function profiles, and a normal complete blood count. TSH was found to be significantly elevated, indicating possible thyroid dysfunction. LH and FSH levels were low, possibly as a result of the patient's prior use of cyclic hormone therapy to regulate her menstrual cycles. We performed a review of the literature, and based on this, a final diagnosis of benign multicystic ovaries, likely attributable to untreated hypothyroidism, was considered. Additionally, the patient was advised to undergo magnetic resonance imaging (MRI) of the pelvis, but the parents opted not to proceed with the procedure due to economic reasons.

The consent form regarding the mode of treatment was duly signed by both the mother and father of the patient, explaining the grave risks of cyst rupture, intra-abdominal hemorrhage, and subsequent danger to her life. The patient was advised exploratory laparotomy and excision of ovarian cysts. However, the parents opted for conservative therapy and desired to avoid surgery. The girl was prescribed levothyroxine 100 mcg, vitamin D, and a ferrous ascorbate tablet. After 20 days, she returned for a follow-up appointment, and a repeat TSH determination was performed. The TSH level now measured 0.052 mIU/ml and she had also lost 3 kg of weight. Considering the TSH level, the levothyroxine dose was reduced to 50 mcg. Transabdominal ultrasonography was performed, showing a reduction in the size of the ovaries; the right ovary measured 92x57x87 mm (242 cc), and the left ovary had dimensions of 99x50x37 mm (106 cc).

The girl was reexamined after one month of therapy, and all her clinical parameters showed immense improvement. She now weighed 59 kg, having lost 6 kg, and she was comfortable with no abdominal pain. TAS was performed, revealing that the left ovary measured 74x72x42 mm (volume 117 cc) and the right ovary measured 51x45x29 mm (volume 38 cc). The cystic ovaries had considerably reduced in size. The girl continued with the same dose of levothyroxine (50 mcg), and a TAS done after two months of therapy showed that the volume of the right ovary had decreased to 49 cc and the left ovary to 17 cc.

**Figure 3:**
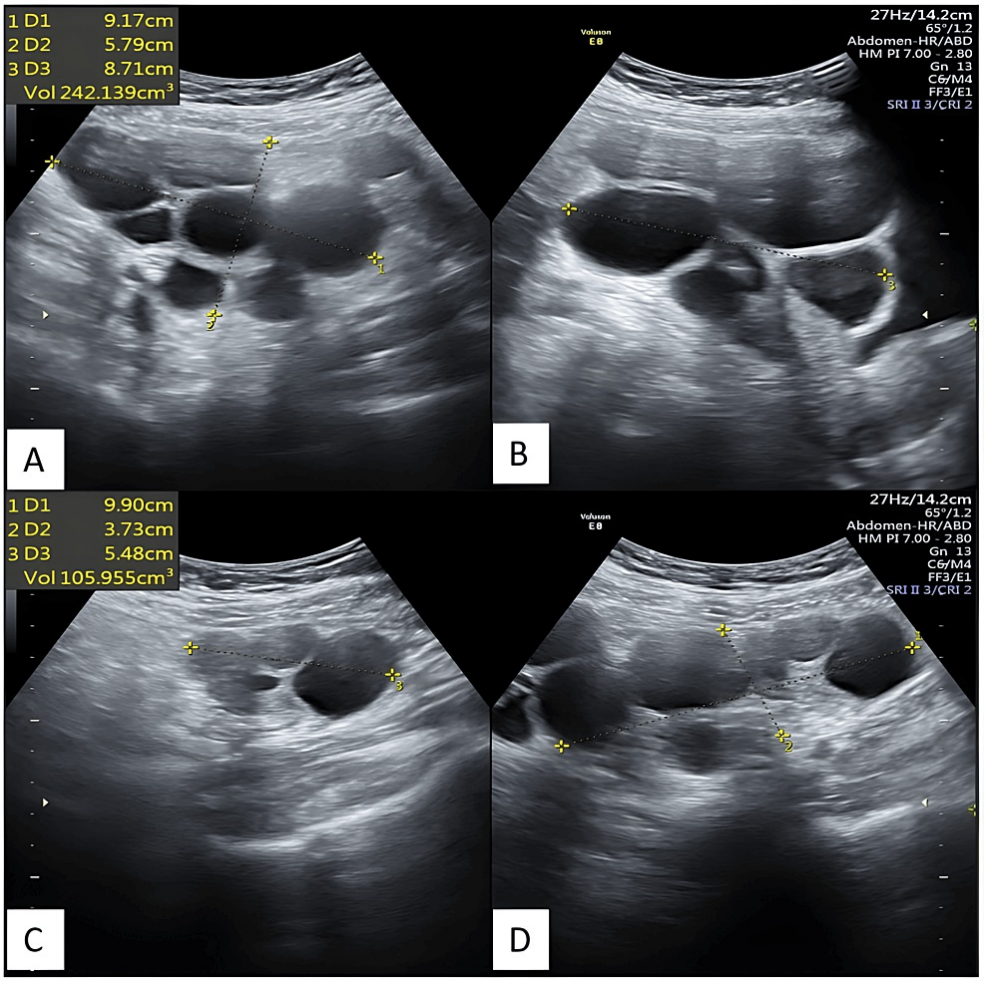
Transabdominal ultrasonography images depict bilateral ovaries with multiple cysts, taken after 20 days of thyroid replacement therapy. The volumes of the ovaries have been reduced compared to the previous scan. Transabdominal ultrasonography reveals an enlarged right ovary in axial (A) and sagittal (B) sections, as well as an enlarged left ovary in axial (C) and sagittal (D) sections, both exhibiting multiple cysts. The volumes of the ovaries have been reduced compared to the previous scan.

Figure 3 depicts transabdominal ultrasonography images taken after 20 days of thyroid replacement therapy, showing enlarged bilateral ovaries with multiple cysts and reduced ovarian volume compared to the previous scan.

**Figure 4:**
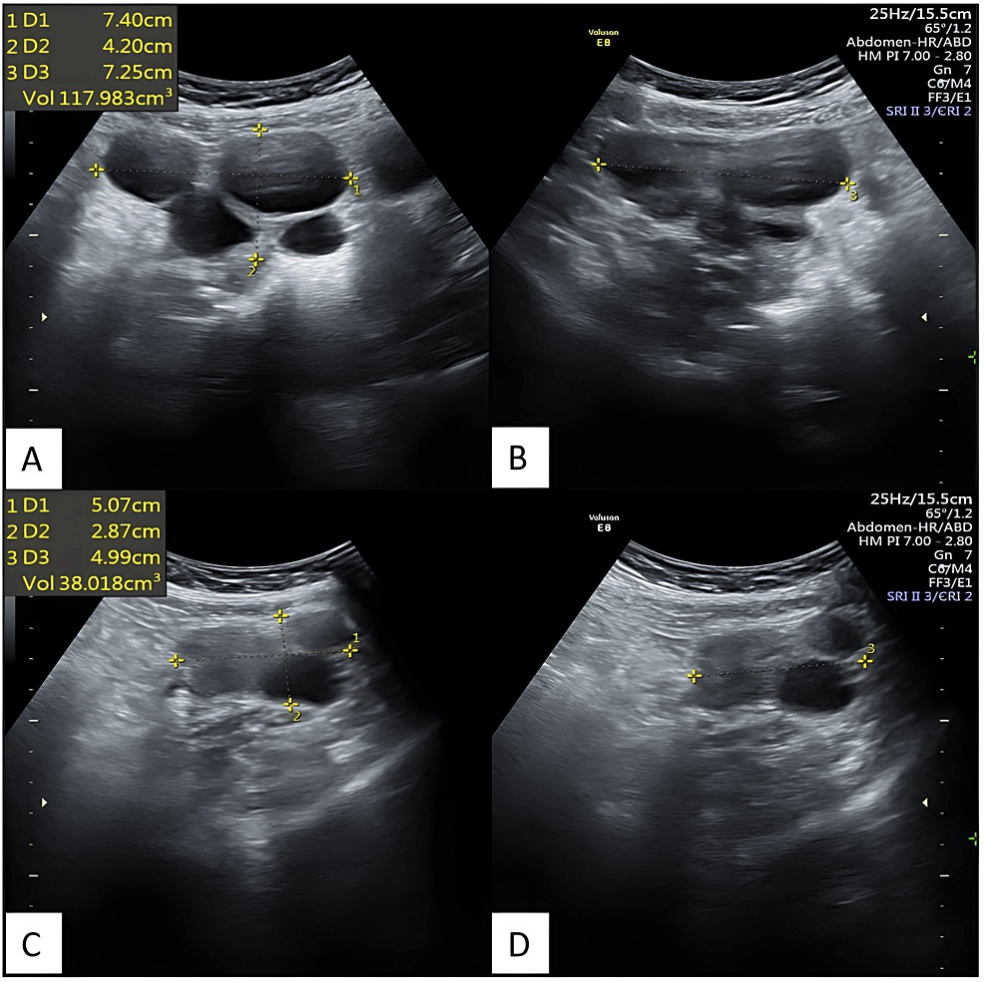
Transabdominal ultrasonography images depict bilateral ovaries with multiple cysts, taken after 34 days of thyroid replacement therapy. The volumes of the ovaries have been reduced compared to the previous scan. Transabdominal ultrasonography reveals an enlarged right ovary in axial (A) and sagittal (B) sections, as well as an enlarged left ovary in axial (C) and sagittal (D) sections, both exhibiting multiple cysts. The volumes of the ovaries have been reduced compared to the previous scan.

Figure 4 depicts transabdominal ultrasonography images after 34 days of thyroid replacement therapy showing enlarged bilateral ovaries with multiple cysts with reduced ovarian volume as compared to the previous scan.

**Figure 5:**
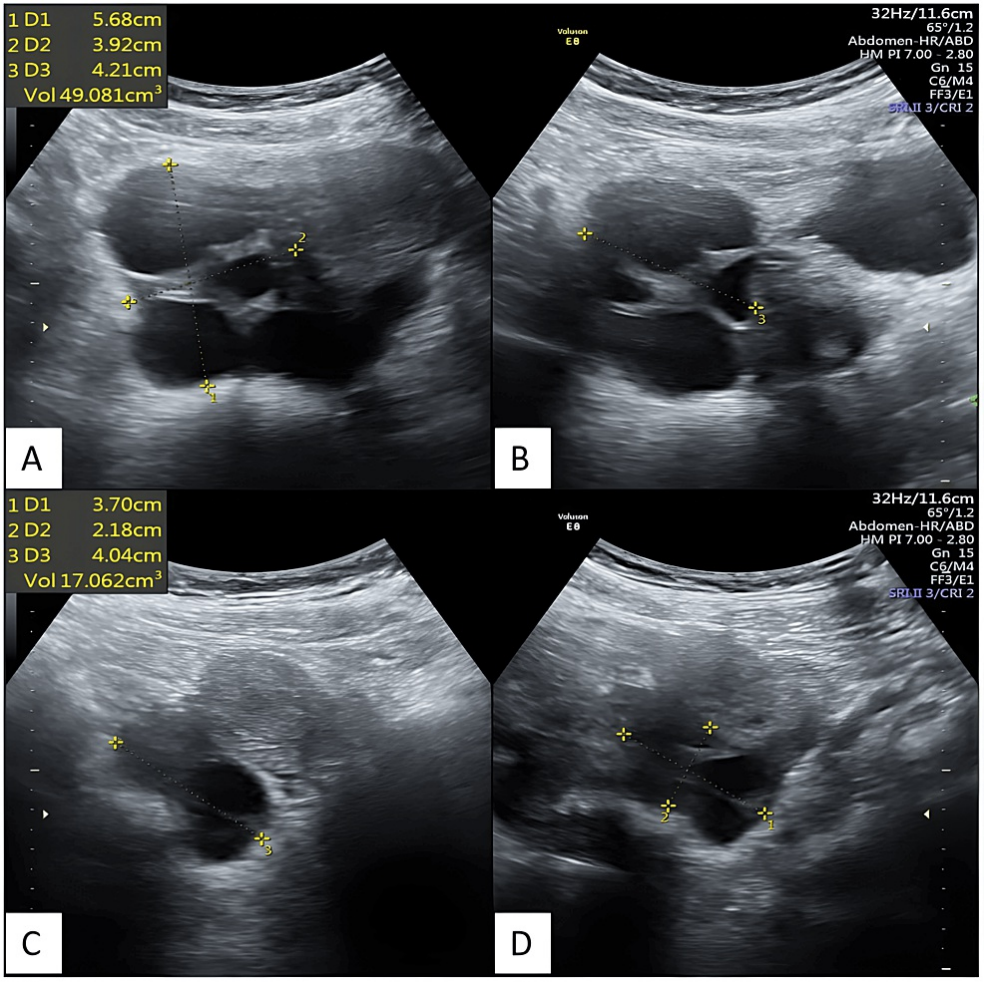
Transabdominal ultrasonography images depict bilateral ovaries with multiple cysts, taken after 67 days of thyroid replacement therapy. The volumes of the ovaries have been significantly reduced compared to the previous scan. Transabdominal ultrasonography reveals an enlarged right ovary in axial (A) and sagittal (B) sections, as well as an enlarged left ovary in axial (C) and sagittal (D) sections, both exhibiting multiple cysts. The volumes of the ovaries have been significantly reduced compared to the first scan.

Figure 5 depicts transabdominal ultrasonography images after 67 days of thyroid replacement therapy showing enlarged bilateral ovaries with multiple cysts with significantly reduced ovarian volume as compared to the first scan.

After one month, the girl was called in for examination and found to have ovaries exhibiting polycystic morphology, with volumes of 15 cc and 12 cc. She began experiencing regular menstrual cycles and appeared cheerful. Her speech now contained substance, and she was responsive to questions, suggesting an improvement in her mental functions. The girl is being called for regular follow-ups at three-month intervals. Her latest TSH level now is 3.4 mIU/ml, and she is currently on 50 mg of levothyroxine therapy. She is now receiving basic educational and vocational training. Simple TSH testing had not been conducted on several occasions, leading to severe mental retardation, poor physical growth, and the development of large ovarian cysts.

## Discussion

The etiopathogenesis of ovarian cyst formation in hypothyroidism was initially suggested by Van Gyk and Grumbach in 1960 [2]. They proposed that TSH, growth hormone, FSH, and LH are all glycoproteins, thus prone to cross-reactivity. Elevated TSH levels mimics FSH and LH activity, resulting in the development of luteinized ovarian follicles, thereby promoting the formation of cysts in the ovaries.

In one recently published case series, Case 1 was a 19-year-old woman who presented with bilateral multiloculated ovarian masses, ascites, raised TSH (TSH 130 mIU/ml), and elevated CA125 levels (CA125 292.0 IU/ml). This girl was administered levothyroxine 200 mcg, and there was complete resolution of the ovarian cysts. Our patient was of a similar age, with a similar multicystic architecture of the enlarged ovaries, and the response to thyroid replacement also occurred in the same manner. Case 2, in this series, was a 32-year-old woman who had undergone radical mastectomy for breast carcinoma, with subtotal hysterectomy done, on tamoxifen, with high TSH levels (TSH 146 mIU/ml) and a multiloculated 7x4 cm ovarian cyst. She was treated with 100 mcg thyroxine, and the cyst resolved after two months of therapy. Case 3, in this series, was a 51-year-old woman with a right-sided adnexal lesion of size 14x10 cm, with solid areas, and a 5x5 cm left-sided ovarian cyst. She also had ascites and a TSH value of 300 mIU/ml. She was given levothyroxine 300 mcg. The ovarian mass subsided completely, and ultrasound after three months revealed normal-sized ovaries [1].

Spontaneous ovarian hyperstimulation due to hypothyroidism has been reported in one case study. In this study, a 15-year-old girl presented with enlarged ovaries containing multiple cysts and mild ascites. The girl was found to have a TSH level of more than 100 mIU/ml. As no other etiology of ovarian hyperstimulation syndrome was detected, it was decided to provide her with levothyroxine at a dose of 100 mcg/day. After four months of therapy, there was complete resolution of the cysts, and normal ovaries were observed [3].

In one research publication, a 14-year-old girl presented with marked asthenia and abdominal pain. She was diagnosed with severe hypothyroidism, characterized by very high TSH levels (960 mIU/ml), and bilateral ovarian cysts. The patient underwent resuscitation, and levothyroxine was immediately administered. Her general condition and biochemical and hormonal assays normalized with therapy. However, on follow-up ultrasonography, her right-sided ovarian cyst did not decrease in size, necessitating wedge resection of the ovary [4]. In our study, the cysts, which were of considerable size, completely regressed in about three months with thyroid replacement therapy.

Other scattered single case reports have been found in further literature reviews, which again suggest that hypothyroidism needs to be considered before resorting to surgery in women with ovarian cysts [5-7]. However, in one case report, a 23-year-old woman underwent left-sided oophorectomy followed by ovarian cystectomy in the right ovary after three years. It was only when the patient presented again, this time with severe malaise, that an endocrine workup was planned. A raised TSH was discovered as the cause of recurrent ovarian cyst formation [8]. A delayed TSH estimation had led to repeated unnecessary surgeries and a significant loss to her ovarian reserve.

After reviewing the literature and presenting our case, we deduce that severe hypothyroidism has a greater propensity for the development of ovarian cysts. Several previously published cases, including our own, have demonstrated the occurrence of large multicystic ovaries in patients with hypothyroidism. Furthermore, our literature review reveals that levothyroxine therapy yields a rapid response, leading to the resolution of cysts within a few months. Consequently, rushing into surgery due to fear of imminent complications should be avoided and deferred.

## Conclusions

A universal practice of testing for TSH at birth, as well as at designated intervals during childhood and early adolescence, would be beneficial in preventing anomalous growth, abnormal pubertal development, and impaired neuro-cognition. The case presentation illustrates that suboptimal thyroid gland function is a significant cause of ovarian cysts, often resulting in cysts of considerable size. Therefore, before considering surgical management for women with ovarian cysts, regardless of their age, a simple and inexpensive TSH determination should always be conducted.
